# Cordycepin Down-Regulates Multiple Drug Resistant (MDR)/HIF-1α through Regulating AMPK/mTORC1 Signaling in GBC-SD Gallbladder Cancer Cells

**DOI:** 10.3390/ijms150712778

**Published:** 2014-07-18

**Authors:** Wei-Ding Wu, Zhi-Ming Hu, Min-Jie Shang, Da-Jian Zhao, Cheng-Wu Zhang, De-Fei Hong, Dong-Sheng Huang

**Affiliations:** Department of Hepatobiliary and Pancreatic Surgery, Zhejiang Provincial People’s Hospital, Hangzhou 310014, China; E-Mails: ZhimingHu11@163.com (Z.-M.H.); shenxiangdi@163.com (M.-J.S.); DajianZhao11@163.com (D.-J.Z.); ChengWuZhangDR@126.com (C.-W.Z.); hongdefei@163.com (D.-F.H.); yishengdongsheng@yeah.net (D.-S.H.)

**Keywords:** the gallbladder cancer, cordycepin, AMPK, multiple drug resistant (MDR), HIF-1α, mTORC1 and chemo-sensitization

## Abstract

Gallbladder cancer is the most common malignancy of the bile duct, with low 5-year survival rate and poor prognosis. Novel effective treatments are urgently needed for the therapy of this disease. Here, we showed that cordycepin, the bioactive compound in genus Cordyceps, induced growth inhibition and apoptosis in cultured gallbladder cancer cells (Mz-ChA-1, QBC939 and GBC-SD lines). We found that cordycepin inhibited mTOR complex 1 (mTORC1) activation and down-regulated multiple drug resistant (MDR)/hypoxia-inducible factor 1α (HIF-1α) expression through activating of AMP-activated protein kinase (AMPK) signaling in gallbladder cancer GBC-SD cells. Contrarily, AMPKα1-shRNA depletion dramatically inhibited cordycepin-induced molecular changes as well as GBC-SD cell apoptosis. Further, our results showed that co-treatment with a low concentration cordycepin could remarkably enhance the chemosensitivity of GBC-SD cells to gemcitabine and 5-fluorouracil (5-FU), and the mechanism may be attributed to AMPK activation and MDR degradation. In summary, cordycepin induces growth inhibition and apoptosis in gallbladder cancer cells via activating AMPK signaling. Cordycepin could be a promising new drug or chemo-adjuvant for gallbladder cancer.

## 1. Introduction

Gallbladder cancer is the most frequent malignancy of the bile duct [1]. It is a highly aggressive and lethal neoplasm associated with high mortality rate and poor prognosis [2,3,4]. Despite recent progresses in diagnostic and therapeutic approaches, the 5-year overall survival is generally low [2]. Due to the absence of specific symptoms and signs, this malignancy is usually detected at an advanced/late stage [2,3,4]. As a matter of fact, relatively few patients with gallbladder cancer are diagnosed prior to surgery [2,3,4]. The surgical resection is the only curative therapy for gallbladder cancer in clinical practices; however, a large proportion of patients will develop recurrences or even metastasis following surgery and/or traditional chemotherapy or radiotherapy [5,6]. Therefore, novel effective therapeutic drugs and adjuvants are urgently needed for this devastating disease.

Cordycepin (or 3'-deoxyadenosine), the bioactive compound present in species of the genus Cordyceps [7,8,9,10], has been shown to exert a large variety of anti-tumor abilities, including cell proliferation inhibition, apoptosis induction, platelet aggregation inhibition, cell migration [7,8,9,10,11,12,13]. However, the potential role of cordycepin in gallbladder cancer cells and the underlying molecular mechanisms are not fully addressed.

AMP-activated protein kinase (AMPK), an evolutionarily conserved serine/threonine protein kinase, is an key energy sensor in all eukaryotic cells [14]. Recent studies have shown that AMPK activation could also promote cancer cell apoptosis and inhibit cell growth [15,16]. As a matter of fact, many anti-cancer drugs including vincristine [17,18], taxol [19,20], temozolomide [21], and doxorubicin [22,23] activate AMPK-dependent cell apoptosis pathway. Further, ursolic acid [24], EGCG [25], quercetin [26] and other anti-tumor plant extracts were shown to inhibit different cancer cells through activating AMPK signalings. Meanwhile, activation of AMPK is important for hydrogen peroxide (H_2_O_2_)-induced apoptosis in cultured neurons [27] and skin keratinocytes [28].

In this report, we investigated the inhibitory role of cordycepin in cultured gallbladder cancer cells and studied the underlying molecular mechanisms. We present evidence that AMPK activation by cordycepin induces mTORC1 in-activation and hypoxia-inducible factor 1α (HIF-1α)/multiple drug resistant (MDR) degradation, which mediates GBC-SD gallbladder cancer cell growth inhibition and apoptosis.

## 2. Results

### 2.1. Cordycepin Inhibits Gallbladder Cancer Cell Survival

**Figure 1:**
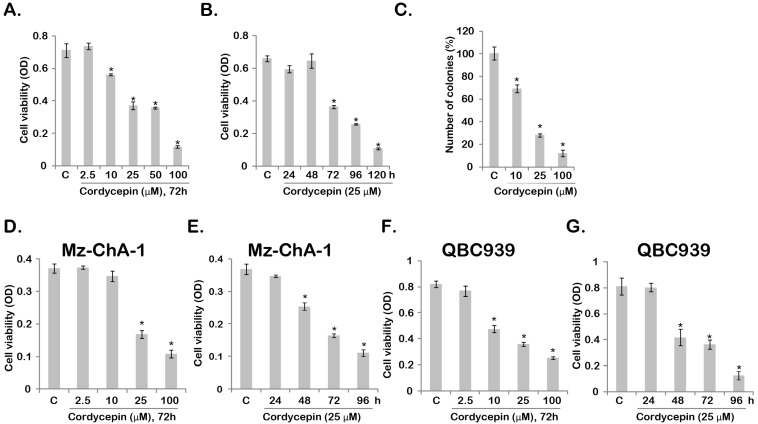
Cordycepin inhibits gallbladder cancer (GBC-SD) cell survival. The viability of GBC-SD cell (**A**,**B**) after indicated cordycepin treatment was tested by MTT assay. GBC-SD cells were cultured in cordycepin-containing medium for 10 days, the number of surviving colonies was recorded (**C**); The viability of Mz-ChA-1 (**D**,**E**) or QBC-939 (**F**,**G**) after indicated cordycepin treatment was tested by MTT assay. * Stands for *p* < 0.05 *vs.* control (“C”) group.

MTT assay was performed to test cordycepin’s effect on gallbladder cancer cell survival. The results in Figure 1A demonstrated that cordycepin dose-dependently inhibited GBC-SD cell viability. Meanwhile, as shown in Figure 1B, the effect of cordycepin was time-dependent, GBC-SD cell viability started to decrease at 72 h after cordycepin (25 μM) treatment (Figure 1B). Further, the results from the clonogenicity assay showed that cordycepin (10–100 μM) significantly inhibited colonies formation of GBC-SD cells (Figure 1C). At the same time, cordycepin was shown to inhibit the survival of two other gallbladder cancer cell lines (Mz-ChA-1/QBC-939, Figure 1D–G), and the effect was once again time- and concentration-dependent. These results show that cordycepin inhibits gallbladder cancer cell survival.

### 2.2. Cordycepin Induces GBC-SD Cell Apoptosis

**Figure 2:**
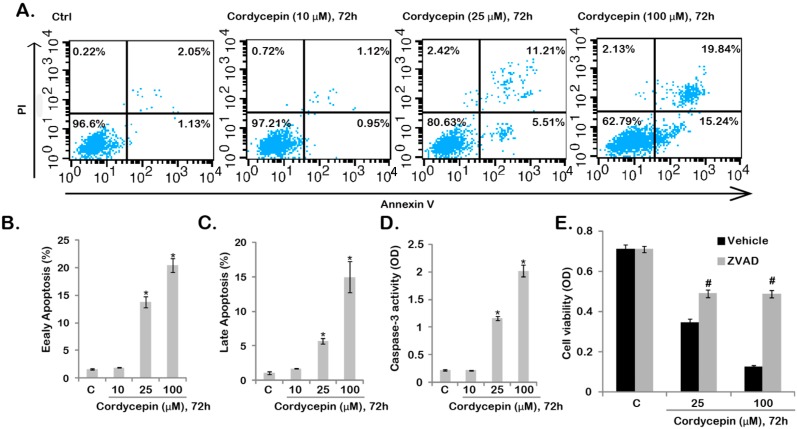
Cordycepin induces GBC-SD cell apoptosis. GBC-SD cells were treated with indicated cordycepin (10–100 μM) for 72 h, cell apoptosis was tested by Annexin V/PI FACS (**A**–**C**) and Caspase-3 activity assay (**D**); Note that for Annexin V FACS assay, the results of five sets of experiments were quantified (**B**,**C**); GBC-SD cells were treated with z-VAD-fmk (50 μM) for 1 h, followed by cordycepin (25/100 μM) stimulation, cells were further cultured for 72 h, and cell viability was tested (**E**); Experiments were repeated five times. * Stands for *p* < 0.05 *vs.* control (“C”) group (**B**–**D**); **^#^** Stands for *p* < 0.05 *vs.* vehicle group (**E**). Vehicle: 0.1% DMSO (**E**).

We then tested the effect of cordycepin on GBC-SD cell apoptosis. Two independent assays including Annexin V FACS and Caspase-3 activity assay were performed to test GBC-SD cell apoptosis after cordycepin treatment. As shown in Figure 2A, the number of Annexin V positive cells was increased dramatically after cordycepin (25–100 μM, 72 h) stimulation, and the effect of cordycepin was dose-dependent (Figure 2B,C). Note that cordycepin induced both early (Annexin V positive/PI negative) and late (Annexin V positive/PI positive) apoptosis of GBC-SD cells (Figure 2B,C). The fact that cordycepin (25–100 μM) increased caspase-3 activity further confirmed apoptosis activation by cordycepin in GBC-SD cells (Figure 2D). As shown in Figure 2E, the apoptosis inhibitor zVADfmk (ZVAD) largely inhibited cordycepin (25/100 μM)-induced GBC-SD cell viability decrease (Figure 2E), suggesting that apoptosis is important for cordycepin-induced survival inhibition in GBC-SD cells.

### 2.3. Cordycepin Inhibits mTORC1 Activation and Downregulates MDR/HIF-1α Expression through Activating of AMPK in GBC-SD Cells

**Figure 3:**
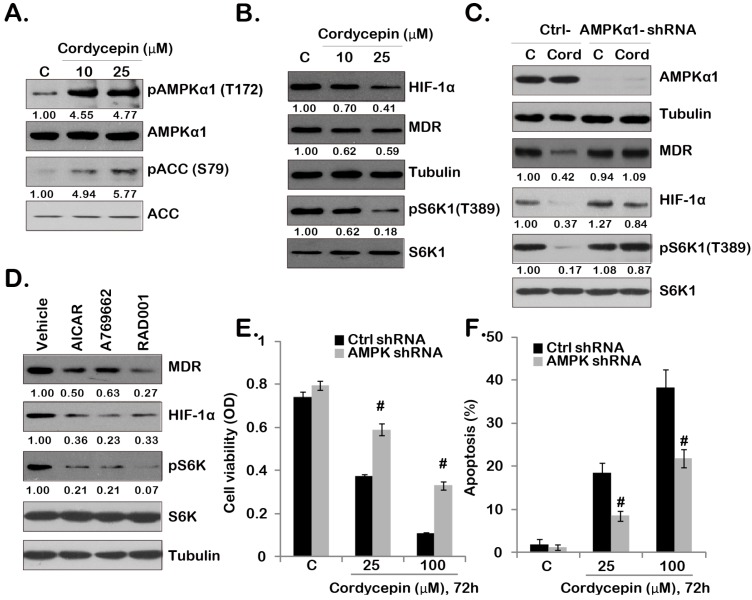
Cordycepin inhibits mTOR complex 1 (mTORC1) activation and down-regulates multiple drug resistant (MDR)/hypoxia-inducible factor 1α (HIF-1α) expression through activating of AMP-activated protein kinase (AMPK) in GBC-SD cells. GBC-SD cells were treated with indicated cordycepin (10/25 μM) for 12 h (**A**) or 24 h (**B**); expression of indicated kinases and proteins was tested by Western blotting (**A**,**B**); The stable GBC-SD cells expressing scramble control short hairpin RNA (shRNA) or AMPKα1 shRNA were treated with cordycepin (25 μM) for 24 h, expression of indicated kinases and proteins was tested by Western blotting (**C**); GBC-SD cells were treated with AICAR (1 mM), A-769662 (10 μM) or RAD001 (500 nM) for 24 h, expression of indicated kinases and proteins was tested by Western blotting (**D**); The viability and apoptosis of stable GBC-SD cells with scramble control or AMPKα1 shRNA after indicated cordycepin treatment were tested by MTT assay (**E**) and Annexin V FACS assay (**F)**, respectively. Experiments were repeated three times. **^#^** Stands for *p* < 0.05 *vs.* scramble shRNA group (**E**,**F**). Indicated bands’ intensity was quantified and normalized to the loading control.

It has been shown that cordycepin activates AMPK signaling in many types of cell, which plays an important role in mediating its biophysical functions [10,13,29]. As shown in Figure 3A, cordycepin-induced significant AMPK activation in GBC-SD cells, as p-AMPKα and p-ACC increased dramatically after cordycepin (10/25 μM) stimulation (see Figure 3A quantification). One of the consequences of AMPK activation is mTORC1 in-activation [30], which could explain why we detected the reduced S6K1 activation (Thr 389 phosphorylation) by cordycepin in GBC-SD cells (Figure 3B). Importantly, cordycepin also downregulated MDR and HIF-1α expression in GBC-SD cells (Figure 3B). As expected, cordycepin-induced S6K1 in-activation was abolished by AMPKα1 shRNA stable knockdown (Figure 3C). Significantly, MDR/HIF-1α down-regulation by cordycepin was also reversed by AMPKα1 depletion (Figure 3C), indicating that AMPK activation is not only required for cordycepin-induced S6K1 inhibition, but is also important for MDR/HIF-1α degradation. To support this hypothesis, we found that AICAR and A-769662, two known AMPK agonists, inhibited S6K1 phosphorylation and downregulated MDR/HIF-1α expression in GBC-SD cells (Figure 3D). Further, RAD001, the mTORC1 inhibitor, induced MDR and HIF-1α degradation (Figure 3D). Based on these data, we suggest that mTORC1 activation is require for MDR and HIF-1α expression in GBC-SD cells, and cordycepin inhibits mTORC1 expression and down-regulates MDR/HIF-1α expression through activating AMPK. Results in Figure 3E,F showed that cordycepin-induced viability loss and apoptosis (combination of early and late cell apoptosis) were largely inhibited in AMPKα1-depleted stable GBC-SD cells, indicating that AMPK activation is also important for cordycepin-induced anti-GBC-SD effect.

### 2.4. Cordycepin Sensitizes Gemcitabine and 5-Fluorouracil (5-FU) Chemo-Response in GBC-SD Cells

**Figure 4:**
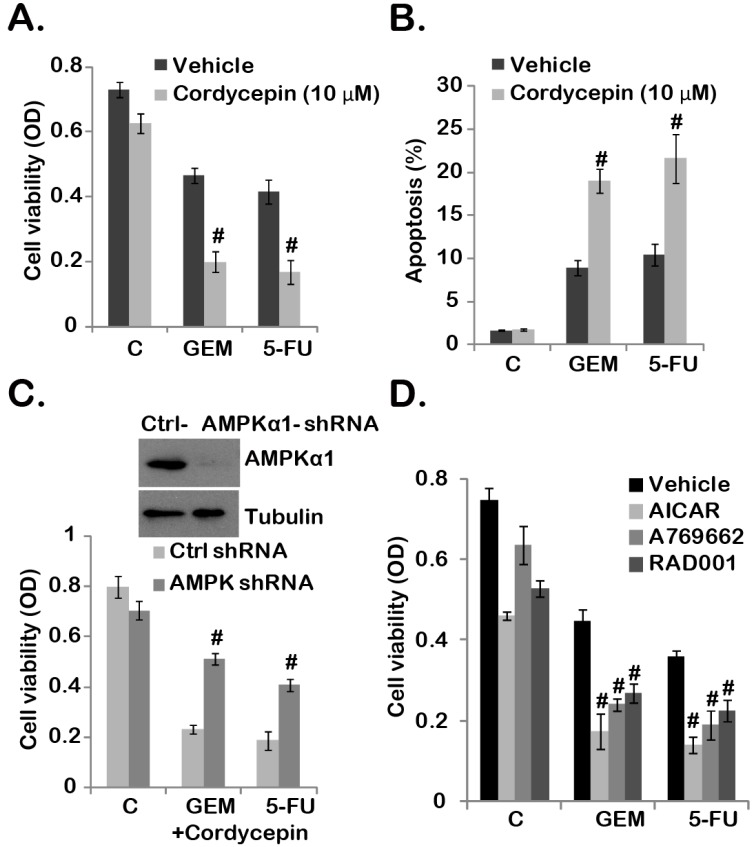
Cordycepin sensitizes gemcitabine and 5-FU chemo-response in GBC-SD cells. GBC-SD cells were treated with indicated gemcitabine (GEM, 0.5 μM) or 5-FU (1.0 μM), in the presence or absence of cordycepin (10 μM) for 72 h, cell viability and apoptosis (combining both early apoptosis and late apoptosis) were tested by MTT assay (**A**) and Annexin V FACS assay (**B**), respectively. Same amount of stable GBC-SD cells expressing scramble-shRNA or AMPKα1-shRNA were either left untreated (“C”), or treated with cordycepin (10 μM) plus gemcitabine (GEM, 0.5 μM) or 5-FU (1.0 μM) for 72 h, cell viability was tested (**C**, **the lower panel**), expression of AMPKα1 and tubulin in stable cells was tested by Western blotting (**C**, **the upper panel**). GBC-SD cells were pre-treated with AICAR (1 mM), A-769662 (10 μM) or RAD001 (500 nM) for 1 h, followed by gemcitabine (GEM, 0.5 μM) or 5-FU (1.0 μM) treatment, cells were further cultured for 72 h, and cell viability was tested (**D**). Experiments were repeated three times. ^#^ Stands for *p* < 0.05 *vs.* vehicle group (**A**,**B**,**D**) or the scramble shRNA group (**C**). Vehicle: 0.1% DMSO (**A**,**B**,**D**).

MDR, HIF-1α and mTORC1 are important chemo-resistance factors [31,32,33], while cordycepin inhibits mTORC1 activation down-regulates HIF-1α/MDR expression (see Figure 3), thus we tested the potential chemo-sensitization effect of cordycepin with gemcitabine or 5-FU, two frequently-used chemotherapeutic agents for human gallbladder cancer treatment [34,35]. Results in Figure 4A,B showed that gemcitabine (0.5 μM) or 5-FU (1 μM) alone only induced moderate viability decrease and apoptosis in GBC-SD cells. However, co-administration with cordycepin significantly increased gemcitabine/5-FU sensitivity (Figure 4A,B). Note that we applied a relative low dose of cordycepin (10 μM), which alone only had a minor effect on GBC-SD cell survival and apoptosis (Figure 4A,B).

The fact that AMPKα1-shRNA depletion largely inhibited cordycepin plus gemcitabine/5-FU co-administration-induced cytotoxicity indicated that AMPK activation was required for the chemo-sensitization effect of cordycepin (Figure 4C). To support this notion, we found that AMPK agonists (AICAR and A 769662) similarly facilitated above chemo-drugs (gemcitabine and 5-FU)-induced viability decrease in GBC-SD cells (Figure 4D). Notably, RAD001, the mTORC1 inhibitor, also enhanced gemcitabine/5-FU-induced GBC-SD cell death (Figure 4D). Based on these results, we suggest that cordycepin increased gemcitabine/5-FU’s sensitivity in GBC-SD cells. AMPK activation appears required for the chemo-sensitization effect by cordycepin.

## 3. Discussion

The mTOR complex 1 (mTORC1), a complex composed of mTOR, Raptor and mSIN1, controls protein translation, cell growth, angiogenesis, and metabolism [36]. MTORC1 over-activation is detected in gallbladder cancer and many other cancers [36,37,38,39]. Activation of mTORC1 results in phosphorylation of a number of downstream targets (*i.e.*, S6K1 and 4E-BP1) involved in cancer progression and apoptosis resistance. MTORC1 activation could induce the mRNA transcription of a number of key oncogenic proteins including cyclin D1 and HIF-1α [36,37,38,39], the latter is a transcription factor that plays a central role in biologic processes under hypoxic conditions. HIF-1α is over-expressed in gallbladder cancer, which is correlated with the poor prognosis [40,41].

Recent studies have shown that AMPK activation could suppress tumor cell growth through in-activating mTORC1 [22,42,43], which is mediated through two following ways: by phosphorylation and activation of TSC2 (Tuberous sclerosis protein 2) [44], the mTORC1 negative regulator [44], and by phosphorylation and inactivating of Raptor (regulatory associated protein of *mTOR*) [45]. In this work, we found that cordycepin inhibited mTORC1 activation and HIF-1α expression in GBC-SD cells, which was almost reversed by AMPKα1 shRNA depletion. Thus, we propose that cordycepin activates AMPK, and in-activates mTORC1 to possibly down-regulate HIF-1α expression in GBC-SD cells.

One of the mechanisms of drug resistance in cancer cells is abnormity of the anticancer drug transport, mediated by members of the ABC superfamily of transport proteins (*i.e.*, MDR) [31]. MDR and other ABC transporters work as efflux pumps to actively decrease the intracellular concentration of anti-cancer drugs [31]. Studies have shown that MDR is over-expressed in gallbladder cancer, and is associated with poor prognosis [32]. MDR depletion/down-regulation could increase the chemosensitivity of gallbladder cancer cells [46]. In this study, we found that mTORC1 activation is important for MDR expression in GBC-SD cells, and RAD001 as well as two AMPK activators (AICAR and A769662) downregulated MDR expression. Further, cordycepin-induced MDR degradation was largely inhibited by AMPK depletion, indicating that AMPK activation by cordycepin inhibits mTORC1 to down-regulate MDR in GBC-SD cells.

The anti-tumor effects of cordycepin have been examined by different groups both *in vitro* and in mice xenografts [47,48,49]. Further, cordycepin could exert anti-angiogenesis ability [48]. Our *in vitro* results in Figure 4 demonstrated that cordycepin potentiated the anti-tumor efficiency of chemo-agents (gemcitabine and 5-FU) in GBC-SD cells [34,35]. The underlying mechanism might be due to cordycepin-induced AMPK activation. Given the facts that gemcitabine and 5-FU are both commonly used anti-gallbladder cancer agents [34,35], and cordycepin, the bioactive compound isolated from Chinese herb, shows low toxicity to mouse normal tissues [48], we propose that cordycepin could also synergize with gemcitabine/5-FU against human gallbladder cancer. However, further studies, including animal studies and possible clinical trials are needed to support this hypothesis.

## 4. Materials and Methods

### 4.1. Chemical and Reagents

Cordycepin, A-769662 gemcitabine, 5-fluorouracil (5-FU) and AICAR (5-amino-1-β-dffff-ribofuranosyl-imidazole-4-carboxamide) were obtained from Sigma (Shanghai, China). RAD001 was obtained from Calbiochem (Darmstadt, Germany). Anti-AMPKα1, HIF-1α, MDR, acetyl-CoA carboxylase (ACC), S6K and tubulin antibodies were purchased from Santa Cruz Biotech (Santa Cruz, CA, USA). Other kinase antibodies (phospho- and regular) used in this study were purchased from Cell Signaling Tech (Denver, MA, USA).

### 4.2. Cell Culture

Mz-ChA-1, QBC939 and GBC-SD human gallbladder cancer cells (all purchased from Shanghai Biological Science Institute, Shanghai, China) were maintained in Dulbecco’s modified Eagle’s medium (Sigma, St. Louis, MO, USA), supplemented with 8% FBS (Sigma), penicillin/streptomycin (1:100; Sigma) and 4 mM l-glutamine (Sigma), in a 5% CO_2_ incubator at 37 °C.

### 4.3. Cell Viability Assay

Cells were seeded at 1.5 × 10^4^ mL cells per well in 96-microculture-well plates. After exposed to the agents as indicated for indicated time, cell viability was assayed using the 3-(4,5-dimethylthiazol-2-yl)-2,5-diphenyl-tetrazolium bromide (MTT) (Sigma, St. Louis, MO, USA) reagent according to the protocol provided. The absorbance was measured at 490 nm.

### 4.4. Analysis of Apoptosis by Flow Cytometry

Cells were collected, washed twice in phosphate buffer saline (PBS), and fixed with ice-cold 70% ethanol for 1 h. The fixed cells were washed and stained with binding buffer containing 50 μg/mL of propidium iodide (PI)/Annexin V, 0.05% Triton X-100, 37 μg/mL of EDTA, and 100 U/mL of ribonuclease. After incubation for 45 min at room temperature, the cell apoptosis is quantified by the flow cytometry with standard optics of FACScan flow cytometer (Becton–Dickinson FACStar, Franklin Lakes, NJ, USA).

### 4.5. Caspase-3 Activity Assay

After treatment, the cytosolic proteins of GBC-SD cells were extracted in hypotonic cell lysis buffer (25 mm HEPES, pH 7.2, 5 mM MgCl_2_, 5 mm EDTA, 5 mM dithiothreitol, 0.05% phenylmethylsulfonyl fluoride). A total of 30 μg of cytosolic extracts were added to caspase assay buffer (312.5 mm HEPES, pH 7.5, 31.25% sucrose, 0.3125% CHAPS) with benzyloxycarbonyl-DEVD-7-amido-4-(trifluoromethyl)coumarin as substrates (Calbiochem, Darmstadt, Germany). Release of 7-amido-4-(trifluoromethyl)coumarin (AFC) was detected, after 1 h of incubation at 37 °C with a fluorescence reader (BD), set to an excitation value of 355 nm and emission value of 525 nm.

### 4.6. Clonogenicity Assay

GBC-SD cells (2 × 10^3^) were suspended in 1 mL of DMEM containing 1% agar (Sigma, St. Louis, MO, USA), 5% FBS and with indicated treatments. The cell suspension was then added on top of a pre-solidified 1% agar in a 100 mm culture dish. The drug-containing medium was replaced every two days. After 10 days of incubation, the left surviving colonies (with diameter larger than 40 μm) were manually counted.

### 4.7. Western Blotting

After treatment, aliquots of 20–30 μg of lysed protein (lysed by 40 mM HEPES (pH 7.5), 120 mM NaCl, 1 mM EDTA, 10 mM pyrophosphate, 10 mM glycerophosphate, 50 mM NaF, 0.5 mM orthovanadate, EDTA-free protease inhibitors (Roche, Basel, Switzerland) and 1% Triton) from each sample were separated by 10% SDS polyacrylamide gel electrophoresis, and transferred onto a polyvinylidene difluoride (PVDF) membrane (Millipore, Bedford, MA, USA). After blocking with 10% instant non-fat dry milk in PBST (the blocking buffer) for 1 h, the membrane was incubated with the specific antibody overnight at 4 °C, followed by incubation with secondary antibody for 2 h at room temperature. The bolt was visualized by ECL (enhanced chemiluminescence) machine. The intensity of the band was quantified through ImageJ software, before normalization to the loading control.

### 4.8. AMPKα1 shRNA and Stable Cell Selection

The AMPKα1 short hairpin RNA (shRNA)-containing lentiviral particles (Santa Cruz Biotech, sc-29673-SH) or the scramble shRNA control lentiviral particles (Santa Cruz Biotech, sc-108080) (20 μL/mL medium each) were added to GBC-SD cells for 48 h, and single stable colony expressing targeted shRNA was selected by puromycin (1 μg/mL). The culture medium (containing puromycin) was replaced every 48 h, until resistant colonies can be formed. The expression of AMPKα1 and equal loading (tubulin) was tested by Western blotting in the resistant colonies.

### 4.9. Statistical Analysis

Statistical analysis was carried out using the SPSS 18.0 software. All values are expressed as the mean ± standard error (SE). Student’s *t*-test was performed to compare the differences between treated groups and their controls. A *p*-value of less than 0.05 was considered statistically significant.

## 5. Conclusions

In brief, the results of this work suggest that cordycepin down-regulates MDR/HIF-1α through regulating AMPK/mTORC1 signaling in GBC-SD gallbladder cancer cells. Further, cordycepin could increase the chemo-sensitivity of known chemo-drugs (*i.e.*, gemcitabine and 5-FU), indicating that cordycepin could be a promising new drug or chemo-adjuvant for gallbladder cancer therapy.

## Abbreviations

AMPK: AMP-activated protein kinase
HIF-1α: 5-fluorouracil, hypoxia-inducible factor 1α
MDR: multiple drug resistant
mTORC1: mTOR complex 1
